# Ambient AI Documentation and Patient Satisfaction in Outpatient Care: Retrospective Pilot Study

**DOI:** 10.2196/78830

**Published:** 2026-02-06

**Authors:** Eric Davis, Sarah Davis, Kristina Haralambides, Conrad Gleber, Gregg Nicandri

**Affiliations:** 1 School of Medicine, University of Rochester Medical Center, 601 Elmwood Ave, Rochester, NY, 14642, United States, 1 585-275-2100; 2 Armstrong Institute for Patient Safety and Quality, Johns Hopkins Medicine, Baltimore, MD, United States; 3 Department of Otolaryngology, University of Rochester Medical Center, Rochester, NY, United States; 4 Department of Medicine, University of Rochester Medical Center, Rochester, NY, United States; 5 Department of Orthopaedic Surgery, University of Rochester Medical Center, Rochester, NY, United States

**Keywords:** ambient AI documentation, Dragon Ambient Experience, DAX, patient experience scores, provider-patient communication, Press Ganey outcomes, artificial intelligence, AI

## Abstract

**Background:**

Patient experience is a critical consideration for any health care institution. Leveraging artificial intelligence (AI) to improve health care delivery has rapidly become an institutional priority across the United States. Ambient AI documentation systems such as Dragon Ambient eXperience (DAX) may influence patient perception of health care provider communication and overall experience.

**Objective:**

The objective of this study was to assess the impact of the implementation of an ambient AI documentation system (DAX) on Press Ganey (PG) patient experience scores.

**Methods:**

A retrospective study was conducted to evaluate the relationship between provider use of DAX (N=49) and PG patient satisfaction scores from January 2023 to December 2024. Three domains were analyzed: (1) overall assessment of the experience, (2) concern the care provider showed for patients’ questions or worries, and (3) likelihood of recommending the care provider to others. Mean pretest-posttest score differences and *P* values were calculated.

**Results:**

A total of 49 health care providers across 9 departments participated in the DAX pilot. Aggregate scores for individual items increased between 0.9 and 1.9 points. Care provider concern for a patient’s questions or worries increased the most (1.9 points; *P*=.01), followed by overall assessment of the experience (1.3 points; *P*=.09) and likelihood of recommending the provider (0.9 points; *P*=.33). Subgroup analysis showed a larger increase in concern scores among providers using DAX <50% of the time (3.2-point increase; *P*=.03).

**Conclusions:**

This pilot study aimed to investigate the relationship between provider use of DAX and PG patient experience scores in the outpatient setting at a large academic medical center. Increases in PG scores after implementing DAX were observed across all PG items assessed. As technology and AI continue to improve and become more widespread, these results are encouraging. Health care providers may consider leveraging AI note-taking software as a way to enhance their communication and interactions with patients.

## Introduction 

### Background

Patient experience is a critical consideration for any health care institution. Understanding the patient experience helps health care institutions continually learn and improve, which supports the delivery of high-quality, patient-centered care [1]. Leveraging artificial intelligence (AI) to improve patient experience and health care delivery has rapidly become an institutional priority across the United States.

### AI Documentation Tools in Clinical Practice

Advances in technology, specifically in AI and natural language processing, have made a tremendous impact on the delivery of health care. There are a number of tools in use that are proven to improve the efficacy of medical providers, decrease administrative burden, and improve work-life balance [2,3]. Recently, there have been considerable advancements in speech-to-text recognition programs that leverage natural language processing and generative AI technology to assist with provider documentation [2,4,5]. Examples include Knowtex, Abridge, and Dragon Ambient eXperience (DAX) [6]. These software products use ambient listening to record the interaction between the health care provider and patient. Through generative AI, they use medically focused large language models to generate a note for provider review and are trained to only include key information succinctly and accurately [2].

It is important to note that medical providers are not the only party impacted by the use of AI in the provision of care. Patients are also vital to consider when deciding to use these tools. For example, facilities using AI tools such as virtual health assistants showed increased satisfaction scores between 2019 and 2021 [3]. Additionally, preliminary research has shown that nearly 20% of adults in the United States expect AI to improve their relationship with their physician and over 30% expect AI to improve their access to care [7].

Using established, validated patient experience metrics can help more comprehensively understand the impacts of AI on the patient experience. One of the largest platforms for measurement of patient experience is the Press Ganey (PG) survey, which is used by over 40,000 hospitals and clinics, leading to over 1 billion patient voices heard [8]. The PG platform allows for measurement and comparison across similar institutions and is relied upon to measure patient experience across the health care industry.

### Study Objectives

Despite the established importance of patient experience in health care, there is a paucity of literature on the impacts of AI use on the patient experience. This study aimed to address this gap by assessing the relationship between provider use of DAX (Nuance) and the patient experience using PG data. Specifically, this study analyzed patient experience scores for providers before and after the implementation of DAX. Our primary hypothesis was that there would be a statistically significant increase in patient experience scores after the implementation of DAX.

## Methods

### Ethical Considerations

This study was determined to be non–human subject research by the institutional review board at the University of Rochester (study 00009626). This retrospective study posed minimal risk and involved no direct participant contact. All data were deidentified before analysis; any potentially identifiable elements (including names or National Physician Identifier numbers) were removed immediately upon access, and health care provider identifiers were removed following data linkage using a temporary crosswalk. Data were stored on secure, password-protected institutional servers, with access restricted to study personnel. Because this study involved retrospective data only and no participants were enrolled or contacted, no compensation was provided.

### Study Design and Setting

We conducted a retrospective study to evaluate the relationship between health care provider use of DAX and patient satisfaction scores as measured using the PG patient experience survey from January 2023 to December 2024.

A total of 49 outpatient care providers participated in the DAX software pilot. The group comprised physicians and nurse practitioners from 9 departments, including internal medicine, family medicine, and orthopedic surgery. Table 1 provides a summary.

**Table 1.** Provider demographics (N=49).

	Participants, n (%)
Health care provider type	
Physician	46 (93.9)
Nurse practitioner	3 (6.1)
Department	
Internal medicine	21 (42.9)
Family medicine	16 (32.7)
Orthopedic surgery	5 (10.2)
Neurology	4 (8.2)
Pediatric medicine	1 (2)
Otolaryngology	1 (2)
Colorectal surgery	1 (2)

### DAX Implementation and Workflow

Patient encounters were recorded using the DAX tool. DAX is an AI documentation tool that allows for automatic documentation of patient encounters through the use of ambient listening and generative AI. Specifically, at the start of a visit, after obtaining consent, the provider accesses the AI documentation tool through their mobile device. Ambient listening is used to record the provider-patient encounter. When the visit is complete, the recording is stopped, and generative AI that uses medicine-specific large language models generates a subjective, objective, assessment, and plan note that is available within seconds. The provider is then able to review, edit, and sign the note into the patient’s record.

### Data Sources

Two primary data sources were used in this study: (1) the number of provider encounters conducted using DAX, which was available in the Epic Signal database; and (2) the patient experience survey from PG. All data were collected from January 2023 to December 2024.

### PG Measures

The PG patient experience survey is voluntary and emailed to all patients following an outpatient visit with a member of the medical faculty group. Patients are administered 1-item measures regarding their experience with the health care provider and facility. Each item is measured on a Likert-type scale from 1 to 5 (1=“very poor”; 5=“very good”). Responses to the following three items were analyzed in this study: (1) overall assessment of the experience, (2) concern the care provider showed for patients’ questions or worries, and (3) likelihood of recommending the care provider to others.

The above items were chosen because of their focus on provider communication and interpersonal quality, which are the domains most likely to be influenced by DAX. For each of the 3 domains, responses were weighted, and a mean score was calculated using the average of all responses. Specifically, the scale from 1 to 5 was converted to a 100-point scale in which “very poor” (1)=0, “poor” (2)=25, “fair” (3)=50, “good” (4)=75, and “very good” (5)=100. Following weighting, the scores were added up and divided by the total number of responses for that domain to assign a score to the provider.

### DAX Use Categories

To evaluate the impact of DAX use on patient experience scores, a pre- vs postuse analysis was conducted. Specifically, patient experience domain scores were calculated before and after the date when a health care provider began using DAX for their encounters. For all providers, the first use of DAX was between March and July 2024. The “before” period was defined from January 2023 until the first date of use, whereas the “after” period was defined from the first date of use until December 2024. In addition to an aggregate pretest-posttest analysis, we categorized by DAX use according to reported percentage of use. Two thresholds were established: <0% to 50% and >50% to 100%.

### Statistical Analysis

To compare group differences, we conducted 2-sample *t* tests (2-tailed) and extracted *P* values using the Satterthwaite approximation for unequal variances. All analyses were considered statistically significant at *P*<.05. Data were analyzed using Stata (version 17; StataCorp) and SAS (version 9.4; SAS Institute).

## Results

### Health Care Provider Characteristics

Of the 49 health care providers included, 46 (94%) were physicians and 3 (6%) were nurse practitioners. The average monthly DAX use in the postuse period was 52.1% and ranged from <1% to 100% in a given month for a provider.

### Pretest-Posttest PG Score Changes

Interestingly, all items exhibited increases in mean scores following the implementation of DAX. The mean score for patients’ overall assessment of the experience increased from 93.7 (SD 8.8) to 95.0 (SD 7.4*; P*=.09). The mean score for the concern that the care provider showed for a patient’s questions or worries increased nearly 2 full points from 94.3 (SD 9.3) to 96.2 (SD 6.2*; P*=.01). Finally, the mean score for the patient’s likelihood of recommending the provider to another individual increased from 94.0 (SD 10.1) to 94.9 (SD 8.9*; P*=.33). These results are summarized in Table 2.

**Table 2.** Press Ganey mean score comparison (January 2023-December 2024).

	Before		After		*P* value
	Survey responses, n	Score, mean (SD)	Survey responses, n	Score, mean (SD)	
Overall assessment	1640	93.7 (8.8)	3034	95.0 (7.4)	.09
Care provider concern for patients’ questions or worries	1643	94.3 (9.3)	3027	96.2 (6.2)	.01
Likelihood of recommending the care provider	1623	94.0 (10.1)	3021	94.9 (8.9)	.33

### Subgroup Analysis by DAX Use

Analyzing the data further, based on DAX percentage of use of <0% to 50% and >50% to 100%, all scores increased in the postuse period regardless of DAX percentage of use. However, the only statistically significant increase was found for care provider concern for the patients’ questions or worries in the 0% to 50% group, exhibiting a 3.2-point increase (*P*=.03). The results of this analysis are summarized in Table 3.

**Table 3.** Pretest-posttest Press Ganey score summary by Dragon Ambient eXperience use (January 2023-December 2024).

Percentage of use	Score before use, mean (SD)	Score after use, mean (SD)	*P* value
Overall assessment			
0% to 50%	92.6 (8.6)	94.1 (7.9)	.26
>50% to 100%	94.2 (9.4)	95.6 (7.1)	.27
Care provider concern for patients’ questions or worries			
0% to 50%	91.9 (10.2)	95.1 (6.4)	.03
>50% to 100%	95.0 (10.4)	96.8 (6.0)	.16
Likelihood of recommending the care provider			
0% to 50%	92.2 (10.3)	93.8 (9.2)	.32
>50% to 100%	95.1 (10.0)	95.5 (8.7)	.78

## Discussion

### Principal Findings

Examining our aggregate results suggests that implementation of DAX has the potential to positively influence PG patient experience scores regardless of the extent of use. Overall, each patient experience domain showed improvement following DAX adoption, with the greatest gains observed in measures related to provider communication and attentiveness. These findings indicate that DAX may support more patient-centered interactions.

### Interpretation in Context of Patient Experience Literature

Patient experience scores are an important quality indicator in health care and an essential consideration for patient-centered care. As discussed, PG offers widely used, validated measures of the patient experience across the health care industry. However, these scores may be difficult to improve on for a variety of reasons. For example, there are potential outside influences on patient satisfaction outcomes that may result in difficulty influencing these scores as they may be outside of the health care providers’ or the organization’s control. These may include patient demographics such as age and sex; the environment in which care is delivered (eg, location of appointment); and factors related to the survey itself, such as the time between when care is received and when the patient completes the survey [9]. Therefore, consideration of influences that are within the health care team’s control becomes even more important.

Although the use of AI in health care is in its early stages, it has shown considerable promise to improve the delivery of health care across numerous specialties [10-12]. AI has been used to interpret imaging studies [13], predict clinically significant outcomes [14], decrease time spent in documentation, and lower burnout scores [15]. However, to our knowledge, there has been no study that comprehensively assesses the patient experience PG scores following the implementation of AI documentation tools in the health care setting. As such, we sought to assess how using this AI tool could allow for a more patient-focused experience as measured using PG scores.

Patient-centered care is focused on giving patients agency in their health care, requiring that providers and all members of the health care team work alongside the patient for effective and safe care [16]. Our results indicated that, in the aggregate analysis, there were statistically significant increases in PG scores after the implementation of DAX. In particular, the scores that increased significantly were those for the concern that the care provider showed for patients’ questions or worries. This suggests that, after implementing the DAX tool, patients perceived an increase in their providers’ communication skills and patient-centered care.

### Impacts of DAX Use Patterns

As noted in the Results section, provider use of DAX varied widely (<1% to 100% of encounters). Interestingly, when health care providers were divided into subgroups, the only statistically significant result was for the 0% to 50% group for 1 item (care provider concern). This finding may indicate that DAX use interacts with patient-provider communication or other related outcomes such as provider burnout. However, given that only 49 providers were included in this analysis, the sample size was likely too small to allow for meaningful subgroup analysis. Future research should aim to assess how AI tool use thresholds could impact patient satisfaction. One possibility is that providers using DAX less frequently may reserve the tool for encounters requiring more detailed communication or emotional engagement, which could amplify the perceived benefit compared with providers who use DAX uniformly across all visits. Intermittent use may also create a clearer contrast between DAX-supported and nonsupported encounters, potentially contributing to the larger observed change in the <50% group.

Additionally, the wide range in monthly DAX use, which averaged 52.1% but varied substantially across providers, may have attenuated the overall effect observed in the aggregate analysis. Variation in how quickly providers adopted the tool and incorporated it into their workflow could contribute to the smaller or nonsignificant changes in outcomes such as likelihood to recommend. This pattern reinforces the importance of evaluating whether more consistent or widespread use leads to greater improvements in patient experience.

### Limitations

There are limitations that should be considered when interpreting these results. First, this was a small-sample pilot study using data from only 49 health care providers. Future research should gather larger samples to allow for more robust statistical significance testing as well as further breakdowns of the data (eg, more stratified subgroups). Furthermore, the cohort included providers from a broad range of specialties, which introduces heterogeneity in clinical workflows and patient populations. This variability limits the ability to draw specialty-specific or subgroup conclusions and should be examined more rigorously in future research. Second, this was a retrospective study, and data availability limited our ability to measure equal pre- and postintervention periods. Future research should systematically measure real-time tool use and these constructs across equal periods to validate these findings. Third, this study did not compare PG scores for providers who were not part of this pilot study, which may be an area for future investigation. Additionally, PG scores were only available at the provider level and could not be linked to individual encounters, preventing an assessment of patient experience specifically for visits in which DAX was used. Future research should examine encounter-level PG outcomes to better isolate the direct impact of DAX exposure. Finally, our sample represents providers from 1 health care system, and generalizability should be investigated in other settings.

### Conclusions

In summary, this pilot study aimed to investigate the relationship between health care provider use of an AI documentation tool, DAX, and PG patient experience scores in the outpatient setting at a large academic medical center. Increases in PG scores after implementing DAX were observed across all PG items assessed, with 1 of the 3 items exhibiting statistical significance. As technology and AI continue to improve and become more widespread, these results are encouraging. While we were not able to determine whether provider communication itself changed, providers may consider leveraging AI note-taking software to support patient experience as it may help facilitate more effective communication and interactions with patients.

## Abbreviations

AI: artificial intelligence
DAX: Dragon Ambient eXperience
PG: Press Ganey
